# Enzyme-Polymers Conjugated to Quantum-Dots for Sensing Applications

**DOI:** 10.3390/s111009951

**Published:** 2011-10-21

**Authors:** Alexandra Mansur, Herman Mansur, Juan González

**Affiliations:** 1 Department of Metallurgical and Materials Engineering, School of Engineering, Federal University of Minas Gerais, Av. Antônio Carlos, 6627, Pampulha, Belo Horizonte/MG, 31.270-901, Brazil; E-Mail: aapiscitelli@uol.com.br; 2 Department of Physics, Federal University of Minas Gerais, Av. Antônio Carlos, 6627, Pampulha, Belo Horizonte/MG, 31.270-901, Brazil; E-Mail: gonzalez@fisica.ufmg.br

**Keywords:** hybrids, quantum dots, functionalized polymer, enzyme sensor, colloids, nanossensor

## Abstract

In the present research, the concept of developing a novel system based on polymer-enzyme macromolecules was tested by coupling carboxylic acid functionalized poly(vinyl alcohol) (PVA-COOH) to glucose oxidase (GOx) followed by the bioconjugation with CdS quantum-dots (QD). The resulting organic-inorganic nanohybrids were characterized by UV-visible spectroscopy, infrared spectroscopy, Photoluminescence spectroscopy (PL) and transmission electron microscopy (TEM). The spectroscopy results have clearly shown that the polymer-enzyme macromolecules (PVA-COOH/GOx) were synthesized by the proposed zero-length linker route. Moreover, they have performed as successful capping agents for the nucleation and constrained growth of CdS quantum-dots via aqueous colloidal chemistry. The TEM images associated with the optical absorption results have indicated the formation of CdS nanocrystals with estimated diameters of about 3.0 nm. The “blue-shift” in the visible absorption spectra and the PL values have provided strong evidence that the fluorescent CdS nanoparticles were produced in the quantum-size confinement regime. Finally, the hybrid system was biochemically assayed by injecting the glucose substrate and detecting the formation of peroxide with the enzyme horseradish peroxidase (HRP). Thus, the polymer-enzyme-QD hybrid has behaved as a nanostructured sensor for glucose detecting.

## 1. Introduction

For millions of years Nature has wisely designed biomolecules for executing very specific functions in most life forms. Each of them is intrinsically related to the others in complex cascade sequences and pathways, frequently with several reactions occurring in parallel. In the field of nanobiotechnology, increasing demands are created every day searching for new sensing tools and devices for clinical applications, like tissue imaging, vaccines, *in vivo* diagnostic tools, and so forth. The most common strategies adopted by the researchers is the use of naturally occurring biomolecules such as proteins, polysaccharides, and nucleic acids that are very specific in their functions and therefore interesting candidates to be conjugated with different materials. Among several alternatives of biomolecules, enzymes have been frequently chosen as active biosensing molecules due to their specificity, affinity, limit of detection, responsiveness, relative chemical and thermal stability, availability, at reasonable cost compared to other options such as immunoglobulins and polynucleotides [1–4]. Furthermore, the possibility of joining natural molecules with synthetic ones is a relatively unexplored realm of research. By combining biomolecules (*i.e.*, enzymes, proteins, carbohydrates, *etc*.) with synthetic macromolecules, for instance polymers, one can produce a novel class of organic-organic (O-O) hybrid nanostructures and networks aiming to matching designed properties not present in either one separately [5,6]. In biosensors studies, besides the required affinity between the analyte and the bio-component (biological element), the detection process is always a major concern as it should offer specific, fast, accurate and reliable responses. Also, the system has to be biocompatible, non-toxic, integrated and allowing reusability at well-suited costs.

The optical and electronic phenomena have been used for decades as detecting signals in chemical and biochemical reactions. More specifically, fluorescence, an optical emission property, is a powerful tool in biological studies, which relevance relies greatly on the availability of sensitive and selective probes. However, “traditional” chemical fluorophores like organic dyes usually have some disadvantages, such as chemical degradation and instability, photobleaching and usually a broad emission band. On the other side, semiconductor nanocrystals or quantum dots (QDs) have unique tunable properties that turn them promising tools in biological and biomedical researches, nanosensors, photo-electrochemical and light-emitting devices [4,7,8]. Thus, QD-based bio-chemical assays have increasingly drawn the attention of the research and industrial communities and become one of the most exciting grounds in biosensors and nanomedicine diagnosis. The dimension in the nano-order scale of QDs and biomolecules paves the way to design biosensors with a combination of the optical and the electronic properties of QDs and the selective binding and catalytic functions of biomolecules. This subject is fascinating but covering the entire topic is beyond the goal of this paper, and it has been addressed in recent reports and reviews by globally dispersed research groups [9–14].

Since the pioneering work from Clark and collaborators [15] reporting the glucose biosensor, innumerable papers have been published for the detection of a very large number of analytes. Nevertheless, biosensing systems based on the enzymatic reaction of β-d-glucose (referred as substrate) with glucose oxidase (GOx) have been often utilized as a model that could be extended and applied in more bioanalytical methods and different substrates [1]. In addition, glucose is far the most important energy source in human metabolism, and the concentration of glucose in blood is considered as a relevant indicator to human health conditions. The development of reliable methods for monitoring blood glucose has long been an interesting topic, and this challenge has yet to be overcome. Beyond that, hundreds of millions people worldwide suffer from diabetes mellitus, a group of metabolic diseases characterized by high blood glucose levels, which result from defects in insulin secretion, or action, or both [4]. Patients suffering from diabetes mellitus must monitor and control their blood glucose levels to avoid long term damage to organs, coma or even death. In that sense, the tight control of blood glucose level by the diabetic sufferer is of paramount importance and biosensing devices comes to play a key role [3].

Therefore, quantum dots with their exceptional optical and spectroscopic features bring a window of opportunity to be explored as fluorophores in numerous biosensors applications. Quantum dots can be conjugated to biomolecules acting as biomarkers with excellent chemical stability, narrow fluorescence band and high quantum yields [16–18]. Various methods for synthesizing QDs have been proposed, but the large majority was based on high temperature organic routes, such as the chemical trioctyl phosphine/trioctyl phosphine oxide (TOP/TOPO) capping method which have excluded them from being directly used in biological environments [11,14,19–21]. A common way to overcome this limitation has been the ligand-exchange method yielding water-soluble nanoparticles, but generally trough complex and costly processes. Up to now, very few studies have successfully reported the synthesis of water-soluble QDs via entirely bio-compatible processes using relatively facile aqueous colloidal chemistry [19,22–26].

In summary, inspired by mimicking assemblies found in Nature, the merging of biology and nanotechnology has generated a new and interesting class of functional structures with a host of applications in fields such as biosensors, nanomedicine, molecular biology, and biochemistry. The preparation of nanomaterials in which the inherent functionality of “tailored” hybrid macromolecules conjugated to optically active quantum dots can be controlled at nanoscale level is a rather attractive model.

In this paper, we provide results from a proof-of-concept study of the synthesis and characterization of enzymatic biosensor response based on novel hybrid functional nanomaterials. To the best of knowledge, despite several studies reported in the literature dealing with chalcogenides quantum dots, none of them have successfully produced and extensively characterized CdS quantum dots capped by hybrid macromolecules (polymer-enzyme), designed to behave simultaneously as a fluorophore and an affinity-based enzyme via one-step aqueous colloidal route. So, it offers a field with almost unlimited opportunities for investigating these novel hybrid systems as biosensors.

## 2. Experimental Section

All reagents and precursors: thioacetamide (Sigma-Aldrich, Cat#163678, >99%), cadmium perchlorate hydrate (Aldrich, Cat#401374, CdClO_4_.6.H_2_O), sodium hydroxide (Merck, Cat# 1.06498.1000, >99%) were used as received, without any further purification. Terpolymer poly(vinyl alcohol-vinyl acetate-itaconic acid) or simply PVA chemically functionalized (PVA-COOH) containing 1.0 mol% carboxylic acid units (itaconic acid moieties) was kindly donated by Kuraray Corporation (Poval KM-118, Viscosity 26.0–34.0 [mPa.s], Mw = 85,000–124,000 g/mol, degree of hydrolysis = 95.5–98.5%). De-ionized water (DI-water, Millipore Simplicity™) with resistivity of 18 MΩ cm was used in the preparation of all solutions.

### 2.1. Preparation Methods of Precursor Solutions

The precursors were prepared as previously reported in our study [22]. Briefly, for the preparation of the sulfur solution (8.0 × 10^−3^ mol L^−1^), approximately 0.0601 g CH_3_CSNH_2_ (thioacetamide) was added to 75 mL of DI-water in a 100 mL flask and homogenized under moderate manual stirring for 10–15 min. Then, the volume was completed to 100 mL with DI-water. This sulfur precursor stock solution was referred as “SOL-A”. Similarly, for the preparation of the cadmium solution (1.0 × 10^−2^ mol L^−1^), approximately 0.4193 g of Cd(ClO_4_)_2_·6H_2_O (cadmium perchlorate) was added to 75 mL of DI-water in a 100 mL flask and homogenized under moderate manual stirring for 10–15 min. Then, the volume was completed to 100 mL with DI-water. This cadmium precursor stock solution was referred as “SOL-B”.

### 2.2. Synthesis of Enzyme-Polymer Conjugates (EPC)—Bioconjugation of Glucose Oxidase to Carboxylated-PVA

**Figure 1.**
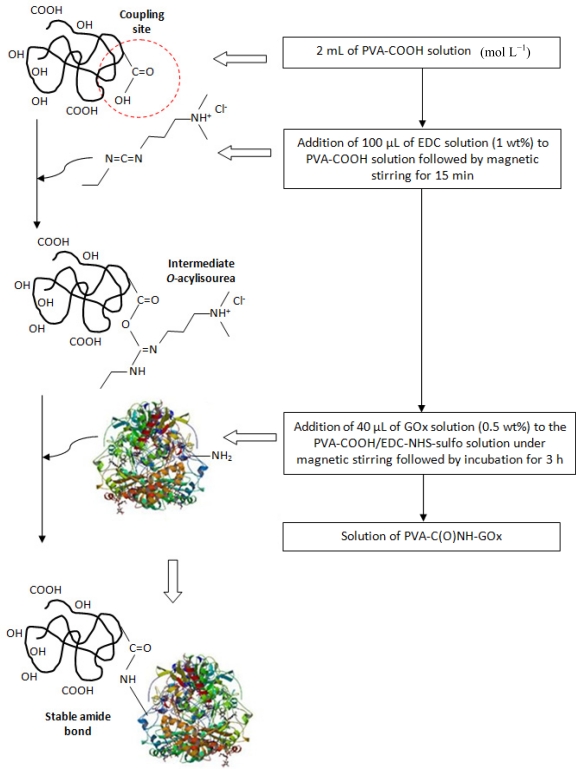
Schematic representation of bioconjugation of the enzyme (GOx) to PVA-COOH producing PVA-C(O)NH-GOx.

The enzyme glucose oxidase (β-d-glucose:oxygen 1-oxido-reductase, GOx, from *Aspergillus niger*, Sigma-Aldrich, PDB entry 1cf3, RCSB Protein Data Bank) was bioconjugated to PVA-COOH using 1-ethyl-3-[3-dimethylaminopropyl]carbodiimide hydrochloride (EDC, Sigma-Aldrich) as “zero-length” crosslinking agent in the presence N-hydroxysulfosuccinimide sodium salt (NHS-sulfo, Sigma-Aldrich). Figure 1 shows a schematic representation of the experimental procedure performed for the bioconjugation of the enzyme (GOx) to the chemically functionalized polymer (PVA-COOH). The bioconjugation process for the glucose oxidase enzyme followed a similar protocol developed for coupling BSA (bovine serum albumin) protein to polymer, reported by our group [22].

PVA-COOH solution (1.0 mol L^−1^) was prepared by adding approximately 4.4 g (PVA-COOH molar unity of ∼44.6 g/mol) to 80 mL of deionized-water in a 250 mL flask. Then, the mixture was heated to 85 ± 5 °C under vigorous magnetic stirring and kept for 4 h when a clear solution was reached. After cooling to room temperature, the solution was transferred into a 100 mL volumetric flask and the volume was completed with DI-water.

EDC (10 mg, 1.0 wt%) and GOx (5 mg, 0.5 wt%) were dissolved in DI-water (1.0 mL). EDC reacts with the carboxyl groups in PVA-COOH forming the amine-reactive *O*-acylisourea intermediate [22]. This intermediate will react with the available amine groups in GOx, yielding a conjugate of PVA-COOH and GOx joined by a stable amide bond (RC(O)NR′R″). This bioconjugated PVA-COOH with GOx was referred as Enzyme-Polymer Conjugate [EPC or “PVA-C(O)NH-GOx”]. The bioconjugated system PVA-C(O)NH-GOx was purified and concentrated using an ultra-centrifugal device with 100,000 M_W_ cutoff cellulose membrane (Amicon filter, Millipore). Centrifugation was conducted for 30 min (6 cycles × 5 min per cycle, at 12,000 rpm, 4 °C) using a Hettich Mikro 200R centrifuge. After the first cycle, it was washed five times with 300 μL DI water. Centrifugal forces caused the removal of the excess of reagents (EDC, M_w_ = 191.7 g/mol; NHS-sulfo, M_w_ = 217.1 g/mol) or unreacted GOx (∼dimer consisting of two equal subunits with a molecular mass of 80 kDa each = 160 kDa) and PVA-COOH through the membrane into the filtrate vial. The resulting conjugate (PVA-C(O)NH-GOx) was resuspended in 500 μL of DI water and stored at 4 °C until further use.

### 2.3. Preparation of CdS Quantum Dots in PVA-COOH and EPC Solutions

CdS quantum dots (QDs) were synthesized via aqueous route in a reaction flask using stock solutions of Cd^2+^ (“SOL-B”) and sulfur (“SOL-A”) precursors, and the previously prepared capping agents, acid functionalized-PVA (PVA-COOH) or EPC (PVA-C(O)NH-GOx). The simplified reaction is represented in Equation (1):

(1) $${\text{Cd}}^{2+}(\text{aq})+{\text{CH}}_{3}C(S){\text{NH}}_{2}(\text{aq})+H_{2}O\to \text{CdS}(s)↓+{\text{CH}}_{3}C(O){\text{NH}}_{2}(\text{aq})+2H^{+}(\text{aq})$$

A typical synthesis was carried out as follows: 2 mL of the macromolecule capping solutions, PVA-COOH or PVA-C(O)NH-GOx, and 45 mL of DI-water were added to the reaction vessel. Under moderate magnetic stirring, the pH was adjusted to (11.5 ± 0.5) with NaOH (1.0 mol L^−1^) slowly added dropwise for approximately 2–5 min. Then, 4.0 mL of cadmium precursor and 2.5 mL of sulfur source solution were added to the flask (S:Cd molar ratio was kept at 1:2). The aqueous system turned yellowish and sampling aliquots of 3.0 mL were collected at different time intervals (1 h, 20 h, and 8 days) for UV-visible spectroscopy measurements that were used for the kinetics analysis and colloidal stability evaluation.

### 2.4. UV-Visible Spectroscopy Characterization of Hybrids

UV-Vis spectroscopy measurements were conducted using Perkin-Elmer equipment (Lambda EZ-210), wavelength from 600 nm to 190 nm, in transmission mode, using quartz cuvettes. The absorption spectra were used to monitor the reaction of CdS formation and relative colloidal stability in the medium. Moreover, based on the “absorbance onset” of the curve it was possible to estimate the average nanoparticles sizes and their optical properties. All experiments were conducted in triplicate (n = 3) unless specifically noted. Statistical analysis was performed assuming the mean and the standard deviation where needed.

### 2.5. TEM/EDS Characterization

The nanostructural characterizations of the CdS-conjugates based on the images and the electron diffraction patterns (ED) were conducted using a Tecnai G2-20-FEI transmission electron microscope (TEM) at an accelerating voltage of 200 kV. The Energy-Dispersive X-ray Spectra (EDS) were collected in the TEM for chemical analysis.

Nanoparticles sizes and distribution data based on the images were conducted using TEM (Tecnai G2–Spirit-FEI, at 120 kV). They were obtained by measuring more than 1,000 individual nanoparticles using the image processing program (ImageJ, version 1.44, public domain, National Institute of Mental Health).

In TEM analyses, samples were prepared by dropping the colloidal aqueous system over a holey carbon grid after the purification step. The purification procedure was carried out using an ultra-centrifugal device with 60,000 M_w_ cutoff cellulose membranes (Amicon filter, Millipore). Centrifugation was conducted for 30 min (6 cycles × 5 min per cycle, at 12,000 rpm) using a Hettich Mikro 200R centrifuge. After the first cycle, it was washed 5 times with 400 μL DI-water. Centrifugal forces caused the removal of the excess of reagents.

### 2.6. Photoluminescence Evaluation in UV “Darkroom-Chamber”

QDs colloidal media were placed inside a “darkroom-chamber” where they were illuminated by UV radiation emission bulb (λ_excitation_ = 245 nm, 6 W, Boitton Instruments). Digital color images were collected as the QDs fluoresce in the visible range of the spectra.

### 2.7. Photoluminescence Spectroscopy Characterization

The emission spectra of the CdS-macromolecule nanohybrids were acquired by using an Ocean Optics USB4000 VIS-NIR spectrophotometer and a Helium-Cadmium (He:Cd) laser at λ = 442 nm (violet-blue, 15 mW of power) as the excitation source. All the photoluminescence (PL) spectra were collected at room temperature.

### 2.8. FTIR Spectroscopy Characterization

FTIR spectra from PVA-COOH and PVA-C(O)NH-GOx films were collected within the 600–4,000 cm^−1^ range (IRAffinity-1, Shimadzu), using the attenuated total reflectance spectroscopy method (ATR-FTIR). Samples were placed onto the ATR crystal prism (ZnSe) and 64 scans were acquired at 2 cm^−1^ resolution with the subtraction of background. Purification of PVA-C(O)NH-GOx solution was carried as describe in Section 2.5 before film casting to remove the excess of reagents.

### 2.9. Sensor Activity of the Bioconjugated Polymer-Enzyme-QD System for Glucose Detection

A biochemical method was utilized for characterizing the synthesis of the enzyme-polymer macromolecule bioconjugates and their behavior as capping ligands for producing CdS quantum dots aqueous sols. Three systems were evaluated: CdS quantum dots capped with PVA-COOH (QD_PVA-COOH used as reference); a “physical” mixture of GOx solution and CdS quantum dots capped with PVA-COOH (QD_PVA-COOH + GOx, Control) and CdS quantum dots capped with the enzyme-polymer conjugate, PVA-C(O)NH-GOx (QD_PVA-C(O)NH-GOx).

The three systems (prepared in triplicate) were filtered in a cellulose membrane (pore size of 0.7 μm) and were washed copiously with DI-water. After that, small pieces of the filter membranes (0.80 cm × 0.80 cm) were cut and positioned at the bottom of the quartz cuvette for the assay.

*Biochemical method:* In this study, the detection of glucose was based on the widely reported protocols using the enzymes reactions in cascade, *i.e.*, GOx and horseradish peroxidase (HRP) [27,28]. In short, the bioconjugation of GOx to PVA-COOH was tested by sensing β-d-glucose with the HRP (Sigma-Aldrich) mediated oxidation of 3,3′,5,5′-tetramethylbenzidime hydrochloride (TMB, Sigma-Aldrich) by H_2_O_2_. The GOx catalyzed oxidation process of glucose produces gluconic acid and H_2_O_2_ [Equation (2)]. Subsequently, the HRP enzyme bio-catalyzed the oxidation of TMB (TMB_ox_, Equation.3). The oxidized specie TMB_ox_ was detected by UV-Vis measurements at different wavelengths (210–215 nm; 290–295; 360–370 nm and 645–655 nm). The overall reactions are shown in Equations (2) and (3):

(2) $$\beta \text{-D-glucose}\;+\;O_{2}+H_{2}O\overset{\text{GOx}}{\to }\beta \text{-D-gluconic acid}\;+\;H_{2}O_{2}$$

(3) $$H_{2}O_{2}+\text{TMB}\overset{\text{HRP}}{\to }{\text{TMB}}_{\text{ox}}+H_{2}O$$

TMB was dissolved in DI-water (1 mg mL^−1^). β-d-Glucose (50 mM) was dissolved in phosphate saline buffer (PBS, pH 7.4). HRP was dissolved in PBS (250 U mL^−1^). The assay of peroxidase activity, which is an indirect assay of glucose oxidase activity, was prepared by adding water (1,188 μL), PBS (600 μL), HRP (12 μL, excess) and TMB (300 μL) and into the 3 mL cuvette containing the filter membrane paper. The experiment was initiated by injecting the glucose substrate (900 μL, final concentration in the cuvette = 15 mM) into the mixture of TMB and HPR in PBS at room temperature. The increase in UV-Vis absorbance at 655 nm was monitored as a function of time (Perkin-Elmer, Lambda EZ-210, wavelength from 800 nm to 500 nm, transmission mode).

## 3. Results and Discussion

### 3.1. Bioconjugation of Enzyme with Polymer

In this work, a totally novel macromolecule was designed and synthesized aiming at combination of physic-chemical and biochemical properties. The acid functionalized polymer (PVA-COOH) was conjugated with the enzyme glucose oxidase (GOx) considered as biomolecule. The reaction of the carboxyl-reactive 1-Ethyl-3-(3-dimethylaminopropyl)carbodiimide (EDC) was utilized as a “zero-length” crosslinker in the presence of NHS-sulfo (catalyst) for covalently coupling the carboxylic group from PVA-COOH to the amines from GOx (protein-NH_2_). Under these conditions, the formation of amide linkages are expected to occur straightforwardly [PVA-C(O)NH-GOx]. The designed system is schematically represented in Figure 1. Besides, EDC has the advantages of not being incorporated into the structure of the protein and not being toxic in comparison with cross-linkers (glutaraldehyde).

**Figure 2.**
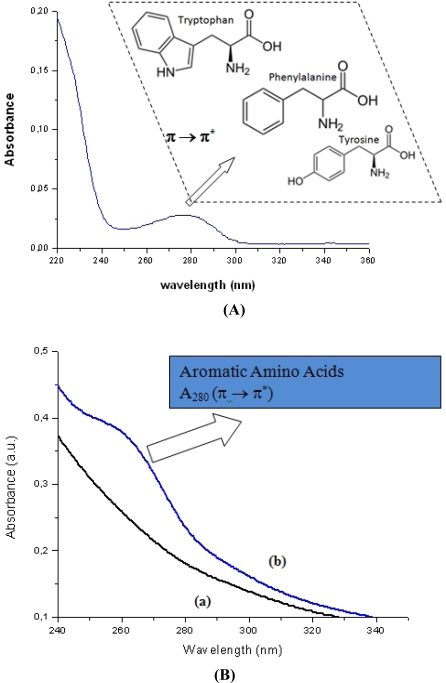
**(A)** UV-Vis spectrum of GOx in aqueous solution used as the reference for the analysis (Insert: electronic transitions (π-π^*^) of cyclic aminoacids). **(B)** UV-Vis spectra of PVA-COOH (curve a) and the EPC system, PVA-C(O)NH-GOx (curve b).

The UV-Vis spectroscopy results are summarized in Figure 2. In Figure 2(A), the UV-Vis spectrum of GOx in aqueous solution used as the reference for the analysis is shown. It can be clearly observed the strong absorption at approximately λ = 280 nm mostly caused by electronic transitions (π-π^*^) of cyclic aminoacids (tryptophan, phenylalanine and tyrosine) present in the enzyme structure. In Figure 2(B) the UV-Vis spectra related to the PVA-COOH (curve a) and the EPC system (PVA-C(O)NH-GOx, curve b) are presented. The significant increase of absorbance in the spectrum of the conjugated PVA-C(O)NH-GOx (curve b) in the region related to π-π^*^ transitions compared to the PVA-COOH not conjugated (curve a) can be observed, which can be assigned to the contribution the enzyme portion of the conjugated system. These findings have indicated that bioconjugation of PVA-COOH with glucose oxidase has successfully occurred.

**Figure 3.**
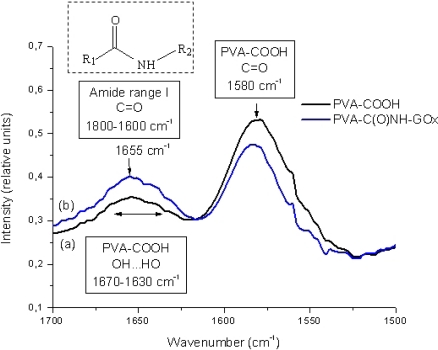
FTIR spectra of PVA-COOH (curve a) and the EPC system, PVA-C(O)NH-GOx (curve b).

FTIR spectroscopy was also performed to characterize in more depth the chemical reaction leading to the formation of the conjugates. The FTIR spectra are presented in Figure 3, with the typical region from 1,500 to 1,700 cm^−1^ related to the formation of amides. The C=O stretching band of carboxyl groups, usually located at 1,750–1,700 cm^−1^, shifts to lower frequencies, mostly due to the ionization of the carboxylic groups, forming carboxylates (COO^−^) with the corresponding symmetric stretch at 1,520–1,610 cm^−1^ [29]. Thus, by comparing the FTIR spectra before [PVA-COOH, Figure 3(a)] and after the conjugation with GOx [Figure 3(b)], the results clearly show a significant reduction in the absorbance region associated with the carboxylate groups (1,500–1,600 cm^−1^) and the simultaneous increase in the amide region (1,800–1,600 cm^−1^) [19]. Hence, based on the UV-Vis and FTIR spectroscopic analyses it was evident that a new artificial macromolecule system was produced by the conjugation of PVA-carboxylic with GOx enzyme.

### 3.2. CdS Quantum Dots—Formation and Stability

**Figure 4.**
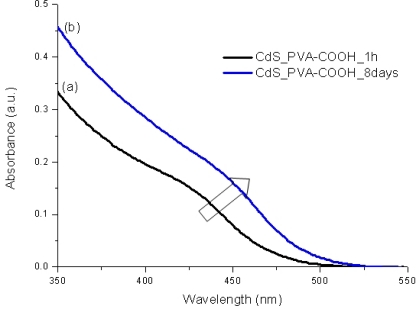
UV-Vis spectra of CdS nanoparticles in PVA-COOH aqueous medium: **(a)** after 1 h preparation; **(b)** 8 days.

**Figure 5.**
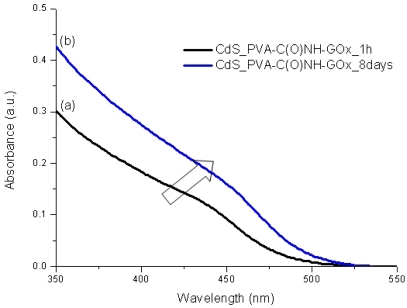
UV-Vis spectra of CdS nanoparticles in PVA-C(O)NH-GOx aqueous medium: **(a)** after 1 h preparation; **(b)** 8 days.

It is widely known that semiconductor nanoparticles made from II–VI elements (*i.e.*, MX, M = Cd, Hg, Pb and X = Se, S, Te) exhibit a very acute change in their optical absorption properties when their sizes are reduced below a certain dimension [30]. Therefore, UV-visible spectroscopy was used to evaluate the effectiveness of new capping agent Enzyme-Polymer Conjugates (EPC) compared to the PVA chemically functionalized with carboxylic groups (PVA-COOH) working as a stabilizer for the formation and growth of CdS nanocrystals in aqueous medium. In Figures 4 and 5 the UV-Vis spectra of the systems after the thioacetamide precursor was added to the PVA-COOH and EPC solutions with Cd^2+^(aq), are shown, respectively.

It can be clearly noted that, for both systems assayed (PVA-COOH and EPC) as the chemical reaction was occurring, there were noteworthy changes in the UV-Vis absorption spectra (“blue-shift”) in the wavelength ranging from 400 to 500 nm, considering from the initial stage [1 h, Figure 4(a), Figure 5(a)] up to 8 days [Figure 4(b), Figure 5(b)]. This behavior can be attributed to the formation and growth of CdS nanoparticles as the reaction time has evolved based on the values of “absorbance onset” from curves a to b (Figures 4 and 5 arrows). Due to the depletion in the concentration of precursors (S^2−^ and Cd^2+^) after some time, the “blue-shift” did not present significant changes, indicating the relative stabilization of the colloidal media. This mechanism for the growth of CdS on the nucleated seeds is represented in [Equation (4)]: [19,22,25]:

(4) $${\text{mCd}}^{2+}(\text{aq})+{\text{mS}}^{2-}\to {\left(\text{CdS}\right)}_{m}+{\text{Cd}}^{2+}(\text{aq})+S^{2-}\to {\left(\text{CdS}\right)}_{m+1}$$

**Figure 6.**
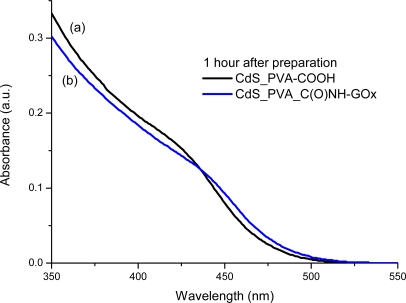
UV-Vis spectra of CdS nanoparticles after 1 h preparation with two ligands: **(a)** PVA-COOH; **(b)** PVA-C(O)NH-GOx.

In addition, from Figures 6 (1 h) and 7 (8 days), it can be noted that both ligands, PVA-COOH and EPC presented similar curves, with a slightly larger “blue shift” for the PVA-COOH. That may be directly related to the CdS nanoparticles kinetics of formation/growth where the comparatively “smaller” ligand, *i.e.*, PVA-COOH, would have had higher mobility in the aqueous media and higher packing density onto the just formed QDs surfaces than EPC as a much larger hybrid macromolecule. Nevertheless, it is feasible to affirm that both PVA-COOH and EPC were effective as capping agents for limiting the semiconductor nanoparticles growth within the “quantum-confinement” dimensional range.

By considering the mechanism of stabilization of colloidal particles in aqueous medium, it may be assumed that the carboxylic moieties in the PVA-COOH polymeric chains were attracted by the “unreacted bonds” of Cd-S at the quantum dots surfaces. The alkaline medium (pH > 11.0) has favored the ionization of the carboxylic groups forming carboxylates (COO^−^). As a consequence, these negatively charged domains would have caused a much stronger attractive force with the positively charged ions (Cd^2+^) at the nanoparticles surfaces. Moreover, the negatively charged polymer competed with sulfides (S^2−^) for metal ion binding sites (Cd^2+^) at the surface and, presumably, to restricting small nanoparticles from aggregating together and growing into larger ones.

A similar trend may also have occurred in the enzyme-polymer ligand as the presence of acid aminoacids residues (*i.e.*, aspartic acid, glutamic acid) in the enzyme portion of the macromolecule would behave quite similarly to the previous described ionic mechanism of PVA-COOH (COO^−^).

On the other hand, for the EPC molecule, as an enzyme-derived system, in the field of biomacromolecules, one should also take into account the presence of localized H-bonding, ionic bonds, hydrophobic interactions of nonpolar groups, van der Waals forces, covalent bonds, crosslinked bonds, sulfide bonds, steric hindrance of active bonding sites, surface chemical groups, substrate surface area, and porosity, just to name but a few. An example of that effect was reported in our recent work where CdS nanocrystals were effectively produced by adsorption with pure protein ligand BSA via aqueous colloidal route [19]. Nevertheless, a full understanding of such systems at the nano-biointerfaces has been a big challenge to the research community in the last few decades, and it out of the scope of this paper [19,31].

### 3.3. Characterization of CdS Quantum Dot Size

#### 3.3.1. Optical Absorption Method

**Figure 8.**
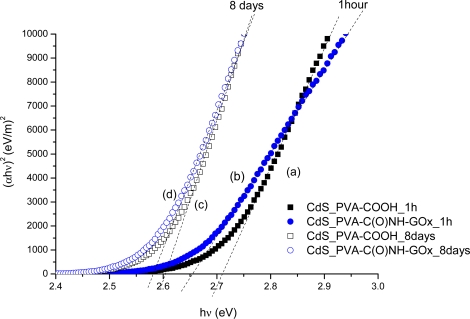
The values of energy band gap estimated during the synthesis of CdS quantum dots with different ligands in aqueous medium: After 1h **(a)** PVA-COOH ligand, **(b)** PVA-C(O)NH-GOx ligand; After 8 days **(c)** PVA-COOH ligand, **(d)** PVA-C(O)NH-GOx ligand.

As briefly mentioned before, semiconductor nanocrystals with the dimensions below the so-called “bohr-radius” will present a quantum-confinement effect, related to the strong interaction between the pair “hole-electron” generated by exciting photon [30]. So, through UV-Vis spectra data, the “absorbance onset” on the curve will be directly related to the altered band gap caused by the quantum-confinement. In essence, in this work, the average nanoparticle size was determined from the Henglein’s empirical model [32] which relates the CdS nanoparticle diameter (2R) to the optical “absorption onset” from UV-Vis spectra according to the Equation (5):

(5) $$2R_{\mathrm{CdS}}\;(\text{nm})=0.1/\left(0.1338-0.0002345\;\lambda_{\mathrm{QD}}\right)$$

where λ_QD_ is the wavelength from the spectral “absorption onset”. The parameter λ_QD_ can be estimated directly from the intersection of the sharply decreasing region of the UV-Vis spectrum with the baseline. However, the assessment of the optical band gap method has been accepted as a more accurate method for estimating the wavelength value (λ_QD_) associated with the “absorbance onset”. Thus, the optical band gap was calculated from absorption coefficient data as a function of wavelength using the “Tauc relation” [33,34], as shown in Equation (6):

(6) $$\alpha h\nu =B{\left(h\nu -E_{\mathrm{qd}}\right)}^{n}$$

where *α* is the absorption coefficient, *hν* is the photon energy, B is the band form parameter, E_qd_ is the optical band gap of the nanoparticles, and n = 1/2 for direct band gap semiconductor as CdS. Therefore, one can estimate the direct band gap value from the plots of (αhν)^2^ *versus* (hν) and extrapolating the straight portion of the graph to (hν) axis, *i.e.*, at α = 0 (dashed lines in Figure 8). Based on the concepts and fundaments described above, it was possible to characterize the formation, stability and average sizes of cadmium sulfide nanoparticles with both ligands, PVA-COOH and EPC. These results are summarized in Table 1. The results have indicated that the CdS QDs were formed at the early reaction stage (1 h) with the estimated diameters of 2.9 nm and 3.2 nm for PVA-COOH [Figure 8(a)] and EPC [Figure 8(b)], respectively. Then, after 8 days they have grown to approximately 3.3 nm [PVA-COOH, Figure 8(c)] and 3.5 nm [EPC, Figure 8(d)]. Similarly, the band gap energy reduced due to the particle growth as the reaction time has evolved.

As expected, a shift to the “red” from the initial band gap energy values (1 h) over 8 days as the result of the CdS nanoparticles’ growth was observed, considering their formation and after reaching stability. These results have provided strong evidence that CdS quantum dots have been effectively produced in the PVA-COOH and EPC media, because the band gap energy values (“blue-shift”) of the semiconductors were always greater than the CdS bulk = 2.4 eV, 2.58 eV (EPC) and 2.61 eV (PVA-COOH) ones. Once again, as discussed in the previous section, the QDs have similar sizes but with a trend to be slightly “smaller” using PVA-COOH as ligand compared to the EPC capping ligand.

#### 3.3.2. TEM Morphological Analysis

**Figure 9.**
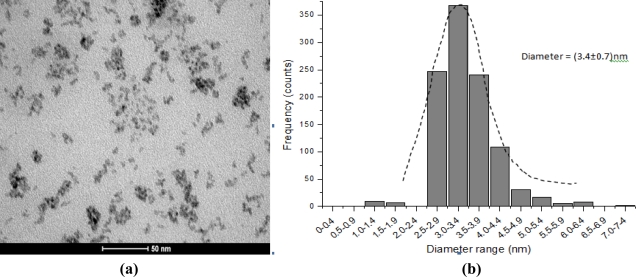
CdS quantum dots capped by bioconjugates PVA-C(O)NH-GOx: **(a)** TEM image and **(b)** particle size distribution histogram.

In this research a complementary evaluation of the quantum dots characterization was carried out using transmission electron microscopy (TEM) for the morphological and structural analyses. Figure 9 shows a representative sample of CdS stabilized with EPC with well-defined dispersed spherical nanoparticles [Figure 9(a)] and an average size of 3.4 ± 0.7 nm [Figure 9(b)]. They have similar dimensions to the PVA-COOH capped nanocrystals previously reported by our group [22]. Thus, TEM results have also proven that CdS quantum dots were properly stabilized by the enzyme-polymer conjugates (EPC) within the quantum-confinement size range and they were consistent with the values estimated by UV-visible spectroscopy in the previous section. Furthermore, it is important to be pointed out that these results regarding to the “total nanoparticles diameters” (CdS and ligand) were below the maximum size recommended for potential biomedical applications, also referred as the “hydrodynamic radius” [22].

### 3.4. Photoluminescence Spectroscopy of CdS Quantum Dots

Despite not being the major goal of the present study, it is known that some other properties are important to be investigated before using such colloidal system as biomarkers. In that sense, photoluminescence (PL) spectroscopy was conducted aiming at verifying the preliminary activity of the CdS nanocrystals produced via aqueous route using a relatively facile method with carboxylic-functionalized PVA as capping agent.

**Figure 10.**
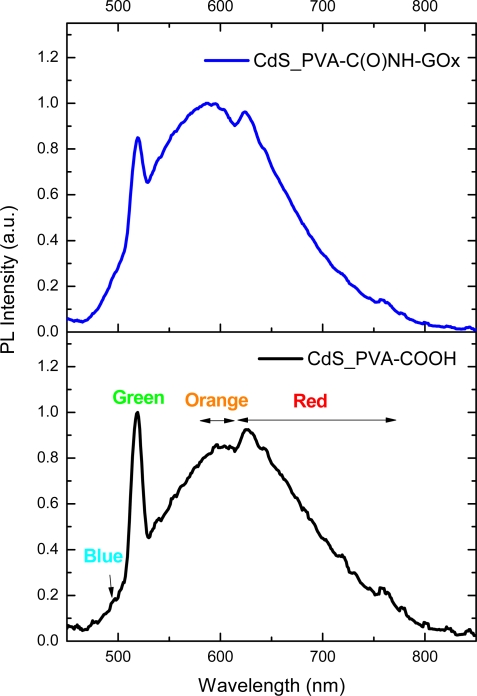
PL spectra for **(a)** CdS_PVA-COOH and **(b)** CdS_PVA-C(O)NH-GOx.

**Figure 11.**
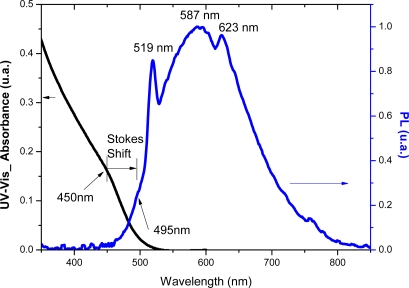
UV-Vis **(a)** and PL spectra for CdS_PVA-C(O)NH-GOx showing Stokes shift of excitonic emission **(b)**.

Figure 10(a) presents a typical PL emission spectrum for CdS nanocrystals synthesized with the carboxylic modified PVA as capping ligand and Figure 10(b) shows the spectrum for CdS prepared in EPC media. It has been reported [35] that crystals of CdS have luminescence in blue, green, orange, and red regions of the spectrum [Figure 10(a)]. Blue luminescence is associated with the excitonic emission and it was observed for CdS_PVA-COOH e CdS_PVA-C(O)NH-GOx at 495 nm overlapped with other emission bands [schematic representation in Figure 10(b)]. These peaks are shifted to higher wavelength regions of PL spectra compared to the position of the absorption band due to the “Stokes shift” (Figure 11). In the “green” region of the spectra, a sharp band at 519 nm was detected in both CdS nanoparticles systems, PVA-COOH and EPC. According to the literature [36,37], this behavior is favored by the synthesis of the nanoparticles under the condition of excess of atoms of the metal that enter in the lattice at interstitial sites (Cd_i_). This assumption is consistent with the experimental procedure utilized in this work, whereby the CdS quantum dots were synthesized using a 2:1 molar ratio of cation to anion, *i.e.*, an excess of metal ions. Orange luminescence (590–625 nm) is observed in cases when interstitial atoms of metal are present in the lattice of semiconductor. So, it is usually detected in crystals with luminescence at about 519 nm. Finally, the red band of PL is typically observed in the nanoparticles which contain a certain concentration of intrinsic defects of the type V_Cd_-V_s_ (di-vacancies) [36].

**Figure 12.**
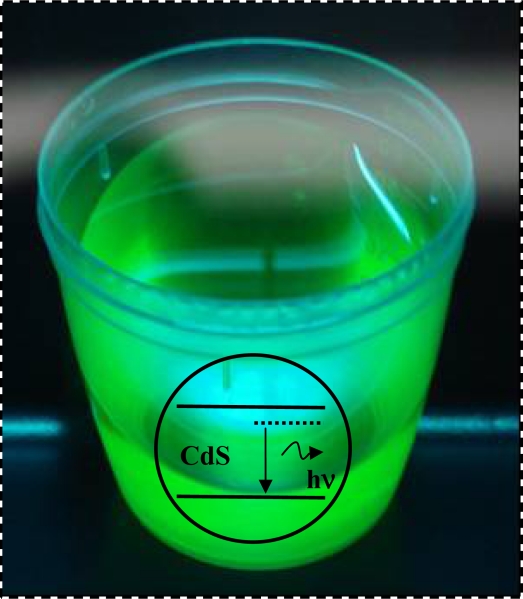
Green luminescence of CdS quantum dots prepared in EPC media (UV lamp, λ_exc_ = 245 nm).

Figure 12 presents the digital image of the synthesized CdS QDs capped with enzyme-polymer conjugates (CdS-PVA-C(O)NH-GOx) with a bright green fluorescence when exposed to ultraviolet light radiation (compartment, light source λ = 254 nm). These results of PL behavior have given incontestable support for considering this novel system based on the hybrid enzyme-polymer-quantum dot (GOx-PVA-COOH-CdS) to be potentially used as active bioconjugates in “optical” sensing and biolabeling applications. Besides that, it brings an environmentally friendly alternative to the labor-intensive method of organic-phase synthesis followed by the ligand exchange.

### 3.5. Sensor Activity of the Bioconjugated Polymer-Enzyme-QD System for Glucose Detection

**Figure 13.**
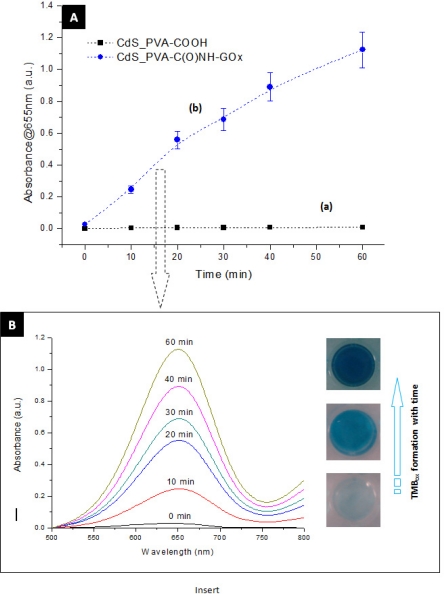
**(A)** Time dependent changes in the absorption at 655 nm of the following systems: **(a)** CdS with PVA-COOH (reference); **(b)** CdS with PVA-C(O)NH-GOx. **(B)** Insert shows the UV-Vis absorption spectra obtained from CdS with PVA-C(O)NH-GOx.

The biosensor activity of the hybrid systems was evaluated and the results are shown in Figure 13.

**Figure 14.**
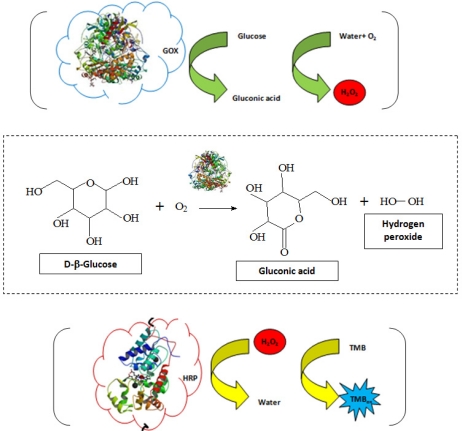
Representative drawing of the biochemical reaction used for evaluating the biochemical activity of GOx conjugation to PVA-COOH.

In Figure 13(A) the biochemical response of the CdS QDs capped by the two ligands, the PVA-COOH and EPC is presented (curves a and b, respectively). The assay was based upon the injection of specific substrate for the enzyme (GOx), *i.e.*, the glucose solution in the system and measuring the spectrometric biochemical response as the reaction time evolved. Thus, it can be clearly observed the sensing activity of the bioconjugated system produced with CdS-EPC compared to the inactive system based on the CdS-PVA-COOH. In Figure 13(B) (insert) the absorbance increase with the time (“blue” color evolution at right images) as a consequence of the reaction taking place catalyzed by the enzyme (GOx) is presented. The developed system utilized the cascade of biochemical reactions [Equations (2) and (3)] for the sensor activity measurements, as schematically represented in Figure 14. The gradual oxidation of the TMB by H_2_O_2_ (formed by the decomposition of glucose) catalyzed by the enzyme horseradish peroxidase lead to the formation of the “blue” chromophore (TMB_ox_) that was detected optically.

It should be said that these results are relevant as far as the enzyme activity is concerned, because the bioconjugation process with PVA-COOH could have caused denaturation or hindered the enzyme catalytic site. Hence, the proposed bioconjugation process in the present study may have altered the 3D conformation of the enzyme due to the chemical covalent bond with the functionalized PVA but has retained its main biochemical activity.

Nevertheless, based on theoretical and experimental aspects, some relevant reduction in the enzyme activity of the bioconjugated system compared to the “free” GOx enzyme activity in aqueous solution should be expected. Essentially, the formation of covalent bonds, steric hindrance, electrostatic forces, hydrophilic and hydrophobic interactions would lead to some decrease in the enzyme activity.

The detection of glucose and/or H_2_O_2_ with the novel enzyme-polymer-QD hybrids was relatively simple and fast. It is worth mentioning that in this study there are two possible “output” signals: optical fluorescence from the QDs (fluorophore) and the chemical product from enzymatic reaction (chromophore), which could be detected once the specific analyte is added into the hybrid system. Also, the system presented relative reasonable detection intensity (absorbance is proportional to concentration, Beer-Lambert’s law) and predictable biocompatibility as QD colloids dispersed in aqueous media.

As a proof of concept, beyond the detection of glucose, this designed CdS-enzyme-polymer nanostructured hybrid has great potential in the development of sensors for various analytes with other biological moieties, for instance targeting peptides, proteins, enzymes, antibodies. In that sense, they can be tailored to a wide range of applications like molecule or cell specific targeting, cell biolabeling and imaging, biocatalyst, drug-targeting carrier, among others [38,39].

## 4. Conclusions

In this study, a novel approach for synthesizing enzymatic biosensors based on hybrid designed functional nanomaterials was presented. CdS quantum dots capped by hybrid polymer-enzyme macromolecules have proven to be simultaneously active as a fluorophore evaluated by photoluminescence and by biochemical enzymatic assay produced via one-step aqueous colloidal route. This may lead to multifaceted biomedical applications such as biosensors at the interface of biology, bioengineering and nanotechnology.

## Figures and Tables

**Figure 7.**
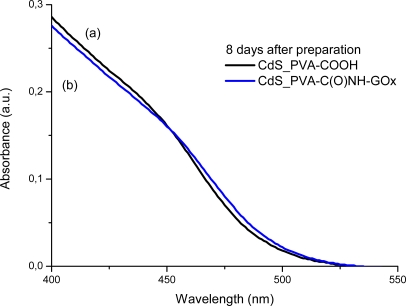
UV-Vis spectra of CdS nanoparticles after 8 days preparation with two ligands: **(a)** PVA-COOH; **(b)** PVA-C(O)NH-GOx.

**Table 1.** Quantum dots parameters: Band-gap energy; blue-shift; estimated particle size.

**Stabilizer**	**Parameter**	**1 h**	**8 days**

PVA-COOH	Band Gap (eV)	2.72	2.61
	Blue Shift (eV)	0.32	0.21
	λ_onset_ (nm)	456	475
	λ_excitonic_ (nm)	423	441
	2R (nm)	2.9	3.3

PVA-C(O)NH-GOx	Band Gap (eV)	2.66	2.58
	Blue Shift (eV)	0.26	0.18
	λ_onset_ (nm)	466	481
	λ_excitonic_ (nm)	437	450
	2R (nm)	3.2	3.5
